# Bridging Crisis and Culture: Comparative Lessons from Chiba and Niigata in Japan’s COVID-19 Health Governance

**DOI:** 10.31662/jmaj.2025-0532

**Published:** 2026-03-27

**Authors:** Kensuke Yoshimura, Kei Tamura, Daisuke Sato

**Affiliations:** 1 Center for Next Generation of Community Health, Chiba University Hospital, Chiba, Japan; 2 Hospital and Health Administration, Fujita Health University Graduate School of Medicine, Toyoake, Japan

**Keywords:** COVID-19, health governance, resilience, Japan

## Abstract

Japan’s COVID-19 response exposed the tension between institutional capacity and human coordination within a highly decentralized health system. Although Japan possesses the world’s highest hospital-bed density among Organization for Economic Co-operation and Development nations, operational capacity proved fragile when infection waves surged. This article employs an integrated comparative design to analyze two divergent prefectural models: Chiba (urban periphery) and Niigata (rural dispersed). By integrating participant-observation analysis from the Chiba Prefectural Task Force with a structured administrative review of Niigata’s administrative records, we identify two complementary governance logics. Chiba demonstrated “Vertical Bridging,” creating new regulatory channels between bureaucracy and private providers to manage resource scarcity. Conversely, Niigata utilized “Horizontal Bonding,” leveraging pre-existing lateral networks, a factor associated with the region’s ability to maintain the lowest mortality rate. These findings challenge the view that physical infrastructure alone dictates resilience, arguing instead that specific governance structures - whether bridging or bonding - significantly influence how capacity can be operationalized. The analysis concludes by detailing how these “soft” assets have been codified into hard mechanisms within Japan’s pandemic measure policies.

## Introduction

The COVID-19 pandemic served as a stress test for Japan’s hybrid health system― a structure characterized by public financing yet predominantly private delivery ^(1)^. While the country boasts 13.2 beds per 1,000 population, the lack of centralized command authority meant that during peak waves, nominal capacity did not translate into operational readiness. Prefectural governments, legally responsible for coordination but lacking direct control over the majority of hospitals, confronted a simultaneous governance and communication crisis.

This paper compares Chiba Prefecture and Niigata Prefecture as critical case studies in pandemic governance. We utilized a purposive sampling strategy based on “Archetypal Sampling” to represent the two dominant archetypes of the Japanese system. Chiba exemplifies the “urban periphery” model, facing acute spillover from Tokyo and high population density. Niigata typifies the “rural dispersed” model, managing logistical fragmentation across a vast geographical area. We explicitly excluded metropolitan outliers such as Tokyo or Osaka, whose exceptional fiscal autonomy renders their models less transferable to the majority of Japan’s 47 prefectures.

Methodologically, this analysis employs an integrated comparative design. For the Chiba case, we utilize participant-observation analysis derived from the authors’ direct participation in task-force meetings (2020-2023). For the Niigata case, we conducted a structured administrative review of primary administrative records, including the “2023 Crisis Response Review” and minutes from 82 Pandemic Headquarters sessions. This approach allows us to cross-verify internal administrative logic against external policy outcomes, identifying the specific governance mechanisms that allowed these prefectures to function as the empirical basis for the Ministry of Health’s 2024 national reform of the Regional Healthcare Plan ^(2)^.

## Chiba Prefecture - Coordination Under Constraint

In early 2020, Chiba Prefecture faced a policy vacuum. The Infectious Disease Control Law mandated the hospitalization of all confirmed cases, yet the region’s medical resources - strained by its proximity to Tokyo - could not support this rigidity. The resulting instability was characterized by a misalignment between public health centers (enforcing mandates) and private hospitals (protecting clinical resources).

To bridge this gap, the prefectural government established the “Hospital Bed Coordination Headquarters”. This body functioned as a mechanism of “Vertical Bridging” - constructing a new regulatory channel between the administrative authority (the Prefecture) and private providers where no effective link previously existed. A team of physicians and civil servants managed daily transfers, translating bureaucratic requirements into clinical reality.

This process revealed the limitations of “onegai-based” governance, a style relying on persuasion, compensation, and professional trust. Lacking coercive power, Chiba’s officials could not compel private compliance. Instead, the Task Force had to construct legitimacy through iterative problem-solving. By documenting recurring bottlenecks - specifically regarding triage criteria and oxygen supply - the Task Force translated field improvisation into standardized protocols.

These “bridging” efforts were instrumental in shaping national policy. The findings were synthesized by the Chiba University-led “Infectious Disease Planning Team”, which proposed quantitative preparedness metrics later adopted in the “8th Regional Healthcare Plan.” Chiba’s experience demonstrated that in the absence of legal command, the state must build “vertical” bridges of data and dialogue to access private capacity.

## Niigata Prefecture - Trust and Transparency as Infrastructure

Niigata’s trajectory offers a distinct contrast. During Japan’s fifth and sixth infection waves, the prefecture achieved the lowest mortality rate nationwide and maintained zero inpatient waitlists ^(3)^. While Niigata’s lower population density (180/km^2^) undoubtedly limits the velocity of transmission compared to urban centers, this geographic advantage was complicated by significant demographic vulnerability. With an aging rate of 32.8% (markedly higher than the national average of 29.1%), the prefecture faced a disproportionately high risk of severe disease requiring hospitalization per infection event. Theoretically, this “aging penalty” should have strained capacity despite lower case numbers. That it did not - and that the region maintained zero waitlists even when infection waves synchronized nationally - suggests structural resilience rather than temporal luck. The outcome reflects superior efficiency in patient flow management, enabled by governance rather than demographics alone.

Niigata operated on the logic of “Horizontal Bonding.” Rather than building new vertical structures, the prefecture leveraged pre-existing lateral networks. The “All Niigata” response was anchored by the Pandemic Headquarters, which convened 82 multi-stakeholder meetings involving medical associations, hospital directors, and municipal mayors. These sessions were not merely bureaucratic updates but transparent strategic forums that generated peer accountability.

Crucially, the “CHAIN Consortium (Niigata Consortium for Healthcare-Associated Infection Control Network)” leveraged horizontal trust. Instead of inspectors, the consortium dispatched peer experts to healthcare and long-term-care facilities, providing hands-on improvements in zoning, Personal Protective Equipment management, and ventilation. This peer-to-peer intervention reduced nosocomial clusters, a primary driver of mortality in aged populations.

Niigata demonstrated that resilience relies as much on “social infrastructure” as physical capacity. By maintaining transparency and utilizing established professional networks, the prefecture converted “latent” trust into “kinetic” operational stability.

While causality cannot be established within the scope of this analysis, the consistency of outcomes across synchronized national waves strengthens the plausibility of governance-related mechanisms.

## Comparative Discussion - Bridging vs. Bonding Governance

Table 1 summarizes the key contrasts between the two governance logics. (Table 1). These categories are presented as ideal-typical analytical lenses rather than mutually exclusive empirical classifications. The experiences of Chiba and Niigata elucidate two distinct operational logics required for decentralized health systems.

**Table 1.** Comparison between Bridging and Bonding Governance.

Prefecture	Chiba	Niigata
Logic	Bridging	Bonding
Mechanism	Learning across fragmented institutions; legal codification of local lessons	Embedded cooperation, transparency, and continuity of leadership
Outcomes	Formal preparedness indicators; integration into national law	Equitable crisis management, high vaccination, minimal excess mortality

1. **Vertical bridging (Chiba):** Required in fragmented urban settings where pre-existing networks are weak or overwhelmed. It involves the state stepping in to create *new* connective tissue between disparate private actors. This model relies on learning―turning ad-hoc crisis responses into formalized rules (e.g., statutory admission criteria).

2. **Horizontal bonding (Niigata):** Effective in settings with strong community cohesion. It involves the state empowering *existing* lateral networks to self-regulate. This model relies on transparency and peer pressure―using shared data to ensure equitable burden-sharing among hospitals.

These distinctions are not merely academic; they define the “architecture of trust.” In Chiba, trust had to be *constructed* through rule-making; in Niigata, trust had to be *activated* through transparency. Both cases refute the notion that the sheer number of beds determines outcomes. In a system where the government owns few hospitals, the unit of resilience is not the facility but the relationship.

## Conclusion - Institutionalizing Trust for Future Preparedness

The pandemic forced Japan to reconcile structural abundance with functional scarcity. The lesson for global health governance is that “trust” cannot remain an abstract virtue; it must be operationalized into administrative mechanisms ^(4)^.

Government policies have codified these lessons, transforming the “soft” assets of Chiba and Niigata into “hard” policy requirements:

•**Incentivizing collaboration:** The Plan introduces the “Infection Control Collaboration Fee,” a financial mechanism that reimburses hospitals for maintaining the “horizontal” networks essential for patient transfer, validating the Niigata model.

•**Mandatory scenarios:** Prefectures are now required to conduct joint tabletop exercises with private hospitals. This institutionalizes the “vertical bridging” seen in Chiba, ensuring that communication channels are tested before a crisis occurs.

•**Data as infrastructure:** The expansion of the Gathering Medical Information System mandates real-time bed visibility, ensuring the transparency that was vital to Niigata’s peer-accountability model.

Ultimately, Chiba and Niigata demonstrate that preparedness is a social contract. Whether through bridging or bonding, the next pandemic will test not the number of beds but the quality of the governance structures that connect them ^(5)^.

## Article Information

### Acknowledgments

The author thanks all the educators and supporters for their dedication to public service. The views and opinions expressed in this article are solely those of the author and do not necessarily represent the positions of the affiliated institutions or of all individuals.

### Author Contributions

Described the data and drafted the manuscript: Kensuke Yoshimura. Participated in the critical revision of the paper: Kei Tamura and Daisuke Sato. All authors have read and approved the final version of the manuscript.

### Conflicts of Interest

None

### IRB Approval Code and Name of the Institution

Not applicable. This manuscript is an Opinion article and does not involve research with human participants or animals.
